# Substance use, affective symptoms, and suicidal ideation among Russian, Somali, and Kurdish migrants in Finland

**DOI:** 10.1177/1363461520906028

**Published:** 2020-03-12

**Authors:** Essi Salama, Anu E. Castaneda, Jaana Suvisaari, Shadia Rask, Tiina Laatikainen, Solja Niemelä

**Affiliations:** 1 Doctoral Programme in Clinical Research, Faculty of Medicine, University of Turku, Finland; 2 Child Psychiatry, Turku University Hospital, Finland; 3 National Institute for Health and Welfare (THL), Finland; 4 Department of Psychology and Logopedics, Faculty of Medicine, University of Helsinki, Finland; 5 Institute of Public Health and Clinical Nutrition, University of Eastern Finland, Finland; 6 Joint municipal authority for North Karelia social and health services (Siun sote), Finland; 7 Department of Psychiatry, University of Turku, Finland; 8 Addiction Psychiatry Unit, Turku University Hospital, Finland

**Keywords:** affective symptoms, binge drinking, cannabis, daily smoking, migrant, suicidal ideation

## Abstract

Comorbidity of substance use with affective symptoms and suicidality has been well documented in the general population. However, population-based migrant studies about this association are scarce. We examined the association of affective symptoms and suicidal ideation with binge drinking, daily smoking, and lifetime cannabis use among Russian, Somali, and Kurdish migrants in comparison with the Finnish general population. Cross-sectional data from the Finnish Migrant Health and Wellbeing Study (Maamu, *n* = 1307) and comparison group data of the general Finnish population (*n* = 860) from the Health 2011 Survey were used. Substance use included self-reported current binge drinking, daily smoking, and lifetime cannabis use. Affective symptoms and suicidal ideation were measured using the Hopkins Symptom Checklist-25 (HSCL-25). We performed multivariate logistic regression analyses, including age, gender, and additional socio-demographic and migration-related factors. Suicidal ideation (OR 2.4 95% CI 1.3–4.3) was associated with binge drinking among Kurds and lifetime cannabis use among Russians (OR 5.6, 95% CI 1.9–17.0) and Kurds (OR 5.5, 95% CI 1.9–15.6). Affective symptoms were associated with daily smoking (OR 1.6, 95% CI 1.02–2.6) and lifetime cannabis use (OR 6.1, 95% CI 2.6–14.5) among Kurdish migrants. Our results draw attention to the co-occurrence of suicidal ideation, affective symptoms, and substance use, especially among Kurdish migrants. These results highlight the variation of comorbidity of substance use and affective symptoms between the different populations. This implies that screening for substance use in mental healthcare cannot be neglected based on presumed habits of substance use.

## Introduction

The comorbidity of substance use with mental health problems and suicidality has been well documented in the general population (Lai et al., 2015; Poorolajal & Darvishi, 2016; Swendsen et al., 2010) and there may be a mutually reinforcing relationship between substance use and mental disorders (Lai et al., 2015; Poorolajal & Darvishi, 2016; Silins et al., 2014; Swendsen et al., 2010). According to Horyniak et al. (2016), substance use is an emerging public health concern among forced migrant populations globally, but epidemiological, population-based research on these issues is still limited (Close et al., 2016; Ezard, 2011; Horyniak et al., 2016; Lo et al., 2017).

A growing body of literature demonstrates that certain migrant populations are at higher risk of mental health problems than the general population (Close et al., 2016; Erdem et al., 2017; Lien et al., 2014). Currently, however, the evidence about suicidal behaviours (suicidal ideation, suicide attempts, or suicide mortality) among migrants is inconsistent. For instance, some studies in Scandinavia have found higher suicidality among migrants in comparison to the general population (Johansson, Sundquist, Johansson, & Bergman, 1997; Johansson, Sundquist, Johansson, Qvist et al., 1997; Patel et al., 2017; Webb et al., 2015, 2016), while others report variation between migrant groups (Hjern & Allebeck, 2002; Johansson, Sundquist, Johansson, Bergman, Qvist et al., 1997; Westman et al., 2003, 2006). In Finland, suicide mortality is lower in migrants compared with the general population (Lehti et al., 2017). Other studies from Europe (Crawford et al., 2005; Goosen et al., 2011; Ikram et al., 2015) and the United States (US) (Brown et al., 2014; Singh & Hiatt, 2006) also found that suicide mortality and suicidal behaviour vary across migrant groups.

Higher prevalence rates of depressive and anxiety symptoms have been reported among Iraqi, Kurdish, Iranian, and Somali migrant populations in comparison with the general populations in various European countries (Bhui et al., 2006; Gerritsen et al., 2006; Siddiqui et al., 2014; Taloyan et al., 2008, 2009). Similar findings have also been reported outside Europe (Ahmad et al., 2016). However, some register-based evidence shows lower prevalence rates among migrants compared with the general population, and thus the current evidence seems to be inconsistent (Markkula et al., 2017). Psychological distress among migrant populations seems to be influenced both by potentially traumatic experiences in the country of origin as well as by hardship and discrimination in the host country (Castaneda et al., 2015, 2017; Tinghög et al., 2007, 2010). In Finland, Kurdish migrants and Russian migrant women have reported higher rates of mental health issues in comparison with the general population (Rask, Suvisaari et al., 2015), high prevalence rates for potentially traumatic experiences have been recorded among Kurdish and Somali origin persons (Castaneda et al., 2017), and high rates of experiences of discrimination have been reported in persons of Kurdish, Somali, and Russian origin (Rask et al., 2018).

Varying traditional habits regarding substance use, different norms, and differences in availability of substances may contribute to differences in substance use among populations worldwide (Wanigaratne, 2018). For example, the prohibition of alcohol and other psychoactive substance use in the Muslim holy book, the Quran, is likely to influence substance use habits in Northern Africa and in the Middle East, where the population is predominantly Muslim (AlMarri & Oei, 2009; Baasher, 1981; Hafeiz, 1995). In contrast, in the Finnish drinking culture, intoxication by alcohol is relatively accepted, and gender differences in alcohol use have decreased in the last few decades (Lindeman et al., 2014; Mäkelä et al., 2012). These cultural factors are likely to contribute to the marked differences in estimated prevalence rates of alcohol use between Western Europe, on the one hand, and North Africa and the Middle East, on the other (Shield et al., 2013). These differences in acceptability and accessibility of alcohol might also influence drinking behaviour after migration.

Substance use habits that are prevalent in the country of origin are theorized to influence the substance use habits of migrants in the new host country (Room, 2004). Currently, the substance use of migrants has been observed to be both more and less prevalent in comparison to the general populations of the new host country, depending on the migrant group, substance, and the general population under study (Abebe et al., 2015; Acartürk et al., 2011; Amundsen, 2012; Carrasco-Garrido et al., 2007; Delforterie et al., 2014; Hjern & Allebeck, 2004; Horyniak et al., 2016; Lindstrom & Sundquist, 2002; Svensson & Hagquist, 2010). However, in Finland, the prevalence of binge drinking among Russian, Kurdish, and Somali origin persons has been reported to be lower in comparison to the general population, whereas daily smoking was more prevalent among Russian and Kurdish migrant men than in the general population (Salama et al., 2018).

Variation in experiencing and expressing psychiatric distress may influence the comorbidity of substance use and mental health across different populations (Kirmayer, 2006; Wanigaratne, 2018). Additionally, the adverse effects of social strain, the cumulative effects of racism and discrimination, as well as the stress from the process of migration and post-migration adjustment, differ between migrant groups (Bhugra et al., 2014; Cantor-Graae & Pedersen, 2013; Gerritsen et al., 2006). These stressors may be internalized into destructive feelings of failure, shame, and rejection, which may lead to impaired mental health and substance use (Walsh et al., 2018).

The existing literature provides some evidence for the association between substance use, suicidal behaviour, and mental health in the countries of origin of migrant populations among these groups. Studies from Russia and Iran indicate that alcohol use and smoking have been associated with poorer mental health (Averina et al., 2005; Ziaei et al., 2017; Zvolensky et al., 2003), suicide attempts (Hooman et al., 2013; Shooshtary et al., 2008), suicidal ideation (Ziaei et al., 2017), and suicide mortality (Pridemore, 2013; Pridemore & Chamlin, 2006). In Iran, illicit substance use has also been associated with worse mental health (Ghaffari Nejad et al., 2011; Nojomi et al., 2007; Poorolajal et al., 2017; Shooshtary et al., 2008; Ziaei et al., 2017) as well as suicide attempts among men (Behmanehsh et al., 2014). Alcohol use has been associated with depressive symptoms among migrants from the former Soviet Union (FSU) in Israel and migrant adolescents of various origins in Norway (Abebe et al., 2015; Massey et al., 2015). Smoking has been associated with psychological distress among Turkish migrants in the Netherlands and Iraqi migrants in Sweden (Erdem et al., 2017; Siddiqui et al., 2014). The studies among Iranian, Iraqi, and Turkish populations did not specify whether persons of Kurdish origin were included in that sample. Comparisons between studies are complicated by varying sample definitions, measures of mental health and substance use, and age groups. Accordingly, very few comparable population-based studies were found.

## Objectives

This study expands on previous findings on the higher prevalence of mental health issues among Kurds and Russian women (Rask, Suvisaari et al., 2015) and the lower prevalence of binge drinking among migrants, but more frequent daily smoking among Russian and Kurdish men (Salama et al., 2018) compared with the Finnish general population. This study aims to determine if affective symptoms and suicidal ideation are associated with substance use among three migrant populations in Finland, and whether the associations are explained by sociodemographic and migration-related factors.

## Methods

### Study design and procedures

The data in this study are from a comprehensive cross-sectional survey, the Finnish Migrant Health and Wellbeing Study (Maamu) (Castaneda et al., 2012), that was carried out by the Finnish National Institute for Health and Welfare (THL) from 2010 to 2012 among populations of Russian, Somali, and Kurdish origin in Finland. The Maamu Study consisted of a health examination and a structured face-to-face interview on health and wellbeing, both conducted by trained bilingual field staff in the participants' native language or in Finnish. The majority of the measures were translated from Finnish into Russian, Somali, and Kurdish (Sorani) and checked by the fieldwork personnel. A short interview was offered to those who were unable to participate in the longer interview. It included the most essential items of the interview and health examination, and was conducted face-to-face, by phone, or by mail as a questionnaire.

### Participants

The survey sample consisted of a stratified random sample of 3,000 persons of Russian, Somali, and Kurdish origin, aged 18 to 64 years, from six big cities in Finland, drawn from the National Population Register. Russian origin was operationally defined as having Russian or Finnish as one's native language and having been born in Russia or the FSU. Somali origin was operationally defined as having been born in Somalia. Kurdish origin was operationally defined having Kurdish as one's native language and having been born in Iraq or Iran. Persons who had been residents of Finland for less than one year were excluded from the sample.

Altogether, the participation rate for the items on affective symptoms, which were collected as part of the health examination, and on substance use, which were collected during in the interview and short interview, was 46% for Russians (*n* = 457), 35% for Somalis (*n* = 350), and 50% for Kurds (*n* = 500). A detailed description of the Maamu Study and its data collection methodology has been reported elsewhere (Castaneda et al., 2012).

Data from the general Finnish population were obtained from the Health 2011 Survey, which was also conducted by the National Institute for Health and Welfare and collected at the same time and with similar methods as the Maamu Study (Koskinen et al., 2012). The comparison group consisted of participants from the same municipalities and the same age group as in the Maamu Study. Data from 860 persons (36%) were obtained on the items of substance use and affective symptoms.

### Ethics

Ethical approval (325/13/03/00/2009) was granted to both studies (Maamu and Health 2011) by the Coordinating Ethics Committee of the Hospital District of Helsinki and Uusimaa. Each participant gave their written informed consent prior to participation.

### Measures

#### Substance use

Binge drinking (no vs. yes) was selected to indicate intoxication-orientated alcohol use. It was probed with the item “How often do you have six or more alcohol units on one occasion?”—with answering categories “never,” “less than monthly,” “monthly,” “weekly,” “daily or almost daily,” from the AUDIT-C questionnaire that was included in the interview (Bush et al., 1998; Dawson et al., 2005; Frank et al., 2008). The variable was dichotomized to binge drinking yes vs. no, where “no” included previous answer “no alcohol use” and answer “never” on binge drinking, and “yes” included answers “less than monthly,” “monthly,” “weekly,” and “daily or almost daily” (Salama et al., 2018). Detailed information on frequency or quantity of alcohol use among those without binge drinking was not available. Therefore, we used dichotomization in order to differentiate binge drinking as a more hazardous alcohol use habit compared to no binge drinking during the previous year.

Daily smoking status was assessed via the question “Do you smoke currently (cigarettes, cigars, pipe)?” with answering categories “yes, daily,” “yes, occasionally,” and “not at all.” No detailed information on frequency or quantity of occasional smoking was available, and therefore we pooled “yes, occasionally” and “not at all.” Thus, daily smoking indicates here a frequent and more hazardous smoking pattern.

Lifetime use of cannabis (yes vs. no) was collected in the interview. No data on cannabis use among the general population were available.

#### Affective symptoms

Affective symptoms are defined here as a mixture of depressive and anxiety symptoms, measured with the Hopkins Symptoms Checklist-25 (HSCL-25) (Derogatis et al., 1974). HSCL-25 has been used to measure clinically significant symptoms of depression and anxiety (Hollifield et al., 2002). It includes 15 items on depressive symptoms and 10 items on anxiety symptoms, probing whether such symptoms occurred during the past seven days; with each item being rated on a scale from 1 “not at all bothered” to 4 “extremely bothered.” HSCL-25 has been used and validated for various populations and its use among ethnically diverse populations has been recommended (Haroz et al., 2016; Hollifield et al., 2002; Tinghög & Carstensen, 2009). The validity of the depression and anxiety subscales has been questioned using the same dataset, and thus the subscales are not used in this article (Kuittinen et al., 2016). Instead, the global scale of HSCL-25 is used to describe overall affective symptoms, similarly to previous research (Gerritsen et al., 2004; Rask et al., 2018; Tinghög & Carstensen, 2009). The dichotomous variable of affective symptoms (yes vs. no) was generated by applying 1.75 as the cut-off mean score for clinically significant symptoms (Gerritsen et al., 2006; Hollifield et al., 2002; Rask et al., 2018; Rask, Suvisaari et al., 2015; Tinghög et al., 2010). HSCL-25 was collected as self-administered questionnaire or by interview with illiterate participants. We used a previously translated version of HSCL-25 (Derluyn et al., 2008).

#### Suicidal ideation

A single item from the HSCL-25 on suicidal ideation, “having thoughts about ending one's life” during the previous seven days, was analysed as a separate item in addition to being included in the HSCL-25. It was examined because of its significance in the clinical context as a severe symptom indicating need for treatment (Goosen et al., 2011; Reko et al., 2015). The item was dichotomized to “no” including the category “not at all” vs. “yes” including categories “a little,” “quite a bit,” and “extremely.” This dichotomized variable was used in the regression analyses.

#### Socio-demographic variables

The socio-demographic variables used were gender, age group (18 to 29; 30 to 45; 46 to 64 years), marital status (married or cohabitating vs. other), level of basic education (secondary school or less, corresponding to the nine years of compulsory education for Finnish citizens vs. higher), employment (employed; unemployed; economically inactive) and a subjective evaluation of the economic situation (satisfactory vs. unsatisfactory). Comparable data on economic situation were not available for the general population. The migration-related variables selected were age at migration to Finland (minor vs. 18 years or more) and self-reported language proficiency in the official languages in Finland (Finnish or Swedish) (good vs. fair or less).

### Data analysis

The statistical analyses were performed using Stata software version 13 IC, and the surveys' sampling design was taken into account in all analyses. We accounted for the effects of missing data using inverse probability weighting, determined by the main predictive factors of nonresponse: migrant group, gender, age, municipality, and marital status (Robins et al., 1994).

The associations of affective symptoms and suicidal ideation with substance use were calculated separately for each subpopulation using univariate logistic regression analysis, adjusting for age and gender. Dichotomized variables of binge drinking, daily smoking, and lifetime cannabis use were used as dependent variables and affective symptoms and suicidal ideation as independent variables. Univariate logistic regression analyses were also conducted separately for both genders. Analyses were conducted also using HSCL-25 as a continuous variable. These results did not differ from those of the dichotomized variables, and thus only the latter are presented in this article. To analyse the effects of the sociodemographic and migration-related factors, multivariate models were created. The variables were selected for the multivariate models based on their association with substance use or with affective symptoms in cross-tabulation (*p* < 0.10), or based on previous results on association (Rask, Suvisaari et al., 2015; Salama et al., 2018). In multivariate analyses, categorized independent background variables were added to the models in a stepwise fashion. A post hoc model was generated to control for the effect of affective symptoms in the association between suicidal ideation and substance use. The odds ratios (OR) and 95% confidence intervals (CI) are reported as age-adjusted figures, and *p*-value < .05 was considered to be statistically significant.

Very few Somali participants had reported substance use and therefore it was not possible to conduct multivariate analyses for Somali participants. Data on lifetime cannabis use of the general population were not available.

## Results

The characteristics of the study sample and the prevalence rates of substance use, affective symptoms, and suicidal ideation are presented in Table 1. The majority of Somalis (71%) and Kurds (75%) had migrated to Finland as quota refugees or asylum seekers, whereas most of the Russians (99%) had another basis for their residence permit (e.g., employment, family ties). Asylum seekers are not registered in the National Population Register until their residence permit is granted, and thus persons still in the process of asylum seeking were not included in the sample. The religion of the majority of Russian origin persons was Christianity (68%), while Somalis and the majority of Kurds (75%) reported to be Muslim.

**Table 1.** Age-adjusted background information on the participants by study population in comparison with the general population.

	Russian			Somali			Kurdish			General population	
	*n*	%	*p*	*n*	%	*p*	*n*	%	*p*	*n*	%
Gender											
Men	161	38	.38	145	43	.67	265	56	< .01	368	41
Age											
18–29	121	30	.97	142	41	< .01	158	34	< .01	55	31
30–45	158	33		144	43		237	47		314	32
46–64	178	37		64	17		105	19		491	37
Marital status											
Married or cohabiting	286	58	.29	228	66	.42	348	67	.21	609	62
Basic education											
High school graduate	364	80	< .01	76	27	< .01	200	42	< .01	557	70
Employment											
Employed	231	49	< .01	68	22	< .01	193	39	< .01	649	62
Unemployed	111	26		82	24		136	27		39	4
Economically inactive	115	26		191	53		169	34		164	34
Economic situation*											
Unsatisfactory	217	48		206	56		329	56			
Age at migration*											
Underage	354	73		210	59		383	74			
Language proficiency*											
Fair or less	192	42		99	23		245	50			
Affective symptoms^1^	79	18	< .01	30	8	.88	176	35	< .01	64	9
Suicidal ideation^1,2,3^	13	3	.48	3	1	< .01	77	16	< .01	29	5
Binge drinking^4^	174	43	< .01	1	0	< .01	78	18	< .01	653	79
Daily smoking^5^	72	16	.75	12	2	< .01	88	19	.16	130	15
Lifetime cannabis use**^,4^	68	17		0			17	4		NA	

*Statistically significant differences (*p* < 0.01) between the migrant groups. **Statistically significant differences (*p* < 0.01) between Russian and Kurdish migrant groups. ^1^Clinically significant affective symptoms ( > 1.75) in HSCL-25 (global scale). ^2^Collected in health examination. ^3^Suicidal ideation during the previous seven days, at least to some extent. ^4^Collected in the interview, but not in the short interview. ^5^Collected in the interview and the short interview.

### Associations between affective symptoms and substance use

**Figure 1.**
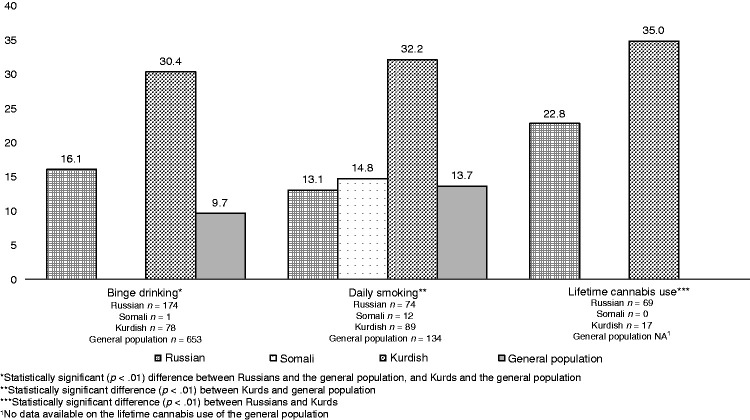
Frequency (%) of affective symptoms among the participants reporting binge drinking, daily smoking, or lifetime cannabis use.

The age- and gender-adjusted prevalence rates of affective symptoms among the participants that reported substance use are presented in Figure 1. Among the participants reporting binge drinking, Russians (16%) and Kurds (30%) reported significantly higher levels of affective symptoms than the general population (10%). Kurds that reported daily smoking had significantly higher levels of affective symptoms (32%) in comparison with the general population (14%).

The associations between affective symptoms and binge drinking, daily smoking, and lifetime cannabis use are presented in Table 2. Affective symptoms were not significantly associated with binge drinking in any study population. Among Kurds, affective symptoms were associated with increased odds for daily smoking and lifetime cannabis use. Among the general population, affective symptoms were associated with daily smoking, adjusting for socio-demographic factors. No significant associations were found among the Russians.

**Table 2.** The association of affective symptoms and suicidal ideation with substance use.

	Model 1^1^				Model 2^2^				Model 3^3^				Model 4^4^			
	OR	95% CI	*p*	*n*	OR	95% CI	*p*	*n*	OR	95% CI	*p*	*n*	OR	95% CI	*p*	*n*
*Binge drinking* ^5^																
Russian																
Affective symptoms	1.2	0.66−2.16	.56	446	1.2	0.63−2.15	.62	444	1.2	0.64−2.32	.55	433	NA			
Suicidal ideation	1.2	0.33−4.00	.82	445	1.2	0.32−4.35	.81	443	1.1	0.25−4.67	.92	432	1.1	0.28−3.99	.93	445
Kurdish																
Affective symptoms	1.3	0.77−2.05	.36	469	1.5	0.93−2.57	.09	460	1.5	0.91−2.52	.11	455	NA			
Suicidal ideation	**2.0**	**1.15−3.38**	**.01**	**467**	**2.5**	**1.38−4.47**	** < .01**	**458**	**2.4**	**1.32−4.33**	** < .01**	**453**	**2.0**	**1.12−3.54**	**.02**	**465**
General population^7^																
Affective symptoms	1.9	0.69−5.25	.22	856	2.3	0.84−6.22	.11	848	NA				NA			
Suicidal ideation	1.9	0.51−7.47	.33	854	1.7	0.44−6.83	.43	846	NA				2.2	0.57−8.82	.25	853
*Daily smoking* ^6^																
Russian																
Affective symptoms	0.8	0.34−1.87	.60	468	0.7	0.25−1.75	.40	454	0.6	0.21−1.51	.25	440	NA			
Suicidal ideation	1.7	0.45−6.75	.42	456	1.7	0.43−6.75	.44	453	1.2	0.26−5.91	.79	439	2.3	0.51−10.42	.28	456
Kurdish																
Affective symptoms	**1.7**	**1.09−2.73**	**.02**	**493**	**1.6**	**1.02−2.62**	**.04**	**482**	1.6	0.99−2.54	.06	461	NA			
Suicidal ideation	1.6	0.92−2.74	.10	491	1.4	0.80−2.42	.24	480	1.3	0.73−2.19	.39	459	1.2	0.66−2.12	.58	489
General population^7^																
Affective symptoms	2.2	0.98−5.15	.06	851	**2.4**	**1.18−4.79**	**.02**	**851**	NA				NA			
Suicidal ideation	**5.18**	**1.55−17.29**	**.01**	**849**	**5.0**	**1.36−17.72**	**.02**	**849**	NA				4.2	0.67−25.83	.13	848
*Lifetime cannabis use* ^5^																
Russian																
Affective symptoms	1.9	0.85−4.06	.12	446	2.0	0.89−4.44	.09	444	1.6	0.70−3.69	.26	433	NA			
Suicidal ideation	**4.8**	**1.52−15.02**	**.01**	**445**	**5.2**	**1.58−17.24**	**.01**	**443**	**5.6**	**1.87−16.96**	** < .01**	**432**	**3.7**	**1.06−12.84**	**.04**	**445**
Kurdish^8^																
Affective symptoms	**6.2**	**2.77−14.14**	** < .01**	**468**	**5.8**	**2.44−13.80**	** < .01**	**459**	**6.1**	**2.56−14.49**	** < .01**	**454**	NA			
Suicidal ideation	**6.0**	**2.53−14.31**	** < .01**	**466**	**5.8**	**2.12−16.09**	** < .01**	**457**	**5.5**	**1.94−15.57**	** < .01**	452	2.9	0.97−8.65	.06	464

Statistically significant findings are in bold. ^1^Adjusted for age and gender. ^2^Adjusted for age, gender, marital status, basic education, employment, and economic situation. ^3^Adjusted for age, gender, marital status, basic education, employment, economic situation, language proficiency, and age at migration to Finland. ^4^Adjusted for age, gender, and affective symptoms. ^5^Somali participants did not report binge drinking or lifetime cannabis use. ^6^Prevalence of daily smoking among Somali participants was too low to perform multivariate analyses. ^7^Without adjusting for economic situation (information not available for the general population). ^8^Age-adjusted with 2-class age variable (18–29; ≥ 30).

### Associations between suicidal ideation and substance use

The associations between suicidal ideation and binge drinking, daily smoking, and lifetime cannabis use are presented in Table 2. Suicidal ideation associated with binge drinking only among Kurds. Suicidal ideation associated with lifetime cannabis use among both Russians and Kurds. Among the general population, suicidal ideation was associated with daily smoking.

### Gender differences

The age-adjusted associations between affective symptoms, suicidal ideation, and substance use examined by gender are presented in Table 3. Among Russians, the association between suicidal ideation and lifetime cannabis use was significant only among women. Among Kurdish men, both affective symptoms and suicidal ideation were associated with binge drinking, but no significant associations were found among women. However, among Kurdish women, affective symptoms were associated with daily smoking, while no significant associations were found among women of other populations or among Kurdish men. Among the women of the general population, suicidal ideation was associated with binge drinking, but no significant associations were found among men of the general population. In contrast, among general population men, both affective symptoms and suicidal ideation were associated with increased odds for daily smoking.

**Table 3.** The age-adjusted associations between affective symptoms, suicidal ideation, and substance use examined by gender.

			Affective symptoms			Suicidal ideation		
		*n*	OR	95% CI	*p*	OR	95% CI	*p*
Binge drinking^1^								
Russian	Men	159	0.9	0.28−3.03	.89	2.6	0.31−21.10	.38
	Women	287	1.4	0.71−2.91	.31	0.8	0.12−5.98	.87
Kurdish	Men	248	**1.9**	**1.10−3.25**	**.02**	**2.4**	**1.27−4.52**	**.01**
	Women	222	0.4	0.11−1.23	.10	1.0	0.28−3.45	.10
General population	Men	367	0.9	0.31−2.94	.93	0.4	0.10−1.41	.14
	Women	498	2.6	0.74−9.45	.14	**4.9**	**1.03−23.14**	**.05**
Daily smoking^2^								
Russian	Men	163	1.1	0.26−4.46	.91	3.3	0.47−23.42	.23
	Women	296	0.7	0.24−1.93	.46	1.3	0.15−11.11	.82
Somali	Men	145	3.1	0.64−14.85	.16	NA		
	Women		NA					
Kurdish	Men	261	1.6	0.95−2.67	.08	1.4	0.76−2.68	.27
	Women	233	**3.6**	**1.08−12.11**	**.04**	2.3	0.80−6.39	.12
General population	Men	366	**5.6**	**2.21−14.05**	** < .01**	**13.5**	**2.24−75.48**	** < .01**
	Women	492	0.9	0.23−3.55	.89	2.3	0.44−11.90	.32
Lifetime cannabis use^1,3,4^								
Russian	Men	160	2.82	0.76−10.51	.12	3.9	0.66−23.48	.13
	Women	287	1.5	0.55−4.08	.43	**4.0**	**1.00−15.83**	**.05**
Kurdish	Men	193	**5.7**	**2.33−13.73**	** < .01**	**5.00**	**1.82−13.73**	**.02**
	Women	NA						

Statistically significant findings are in bold.^1^Somali participants not included due to low prevalence of both affective symptoms and binge drinking/lifetime cannabis use.^2^Somali women not included due to low prevalence of daily smoking.^3^Data on the general population not available.^4^Kurdish women were not included due to low prevalence of lifetime cannabis use.

## Discussion

In this population-based study of Russian, Kurdish, and Somali migrants, we found that affective symptoms and suicidal ideation are intertwined with substance use, especially among Kurds, highlighting the risk of comorbidity. In addition, the associations between substance use and affective symptoms seem to vary between the study populations.

To our knowledge, there are no comparable previously conducted studies that examine the association between suicidal behaviour and substance use among Kurdish migrants or Kurds in general—and suicidal ideation has sometimes been considered a less relevant symptom among predominantly Muslim populations (Kuittinen et al., 2016). Our results on affective symptoms and substance use are mostly in concordance with the existing literature on the general population in Iran (Nojomi et al., 2007; Poorasl et al., 2007; Poorolajal et al., 2017; Shooshtary et al., 2008; Ziaei et al., 2017) and on Iraqi migrants (Siddiqui et al., 2014; Tinghög et al., 2010). Our results on the associations between suicidal ideation and binge drinking/lifetime cannabis use are in line with findings from Iranian city-wide studies on adults and adolescents, where substance use was more prevalent among suicide attempters in comparison to non-attempters (Behmanehsh et al., 2014; Nojomi et al., 2007; Ziaei et al., 2017).

Smoking has been found to be associated with mental health symptoms, psychological distress, and depressive symptoms among Iraqi migrants in Malmo/Sweden and in Turkish migrants, Turkish-Dutch youth, and Moroccan-Dutch youth in the Netherlands (Acartürk et al., 2011; Erdem et al., 2017; Siddiqui et al., 2014). Additionally, in small-scale studies in Iran, current smoking has been associated with psychiatric distress in adult students (Poorolajal et al., 2017), suicidal ideation in adolescents (Ziaei et al., 2017), and was more prevalent among adult suicide attempters (Nojomi et al., 2007; Shooshtary et al., 2008). According to a nationwide study in Iraq, major depression was not associated with substance use disorders. However, the analyses were conducted for the entire population, not distinguishing between Iraqi and Kurdish participants (Al-Hamzawi et al., 2015).

Contradictory to our findings of association between suicidal ideation and binge drinking among Kurds, alcohol use was not associated with mental health symptoms in Iraqi migrants in Sweden or in Turkish migrants in the Netherlands (Erdem et al., 2017; Siddiqui et al., 2014). The differences in findings could be explained by differences in study populations and cultures (Iraqi or Turkish vs. Kurdish), differences in drinking habits in current countries of residence (Sweden and Netherland vs. Finland), or methodological differences (e.g., measures of alcohol use).

Interestingly, our findings on Russian migrants are contradictory to those in previous literature on mental health problems, suicidal behaviour, and substance use among the Russian general population (Averina et al., 2005; Pridemore, 2013; Pridemore & Chamlin, 2006) and in Russian migrants (Averina et al., 2005; Kõlves et al., 2006; Massey et al., 2015). In Russia, hazardous drinking and severe alcohol use have been associated with suicide mortality and suicide risk, especially among men (Pridemore, 2013; Pridemore & Chamlin, 2006). Depressive symptoms have been associated with binge drinking in the Russian general population (Averina et al., 2005) and in migrants from the FSU in Israel (Massey et al., 2015), while smoking has been associated with both depressive symptoms and anxiety/panic disorder in the Russian general population (Averina et al., 2005; Zvolensky et al., 2003). The differences between our results and those in the previous literature could be explained by the voluntary nature of migration of our Russian participants, where the majority had non-humanitarian reasons to migrate (e.g., family ties, work-based migration). Our results on the association between affective symptoms, suicidal ideation, and lifetime cannabis use among persons of Russian and Kurdish origin are in line with previous results (Abebe et al., 2015; Metrik et al., 2016), and add to the existing literature on the association between cannabis use and various mental health symptoms (Lev-Ran et al., 2014; Volkow et al., 2014).

Due to the low prevalence of substance use among Somali migrants in our study, the analyses of associations between affective symptoms and substance use could not be executed. Previously, a low level of substance use (other than khat) has been reported among Somali migrants in the United Kingdom, and khat use has been associated with a higher risk for mental health disorders, suicidal ideation, and cigarette smoking among Somali migrants in London (Bhui et al., 2003, 2006).

According to our results, the associations between suicidal ideation or affective symptoms and substance use were not explained by sociodemographic or migration-related factors. Further, the associations between affective symptoms, suicidal ideation, and substance use varied between the migrant groups, and between genders within migrant groups. Russian participants in our study seem to be a group of mainly voluntary migrants that have not been forced to leave their country of origin. Instead, they have migrated for personal or work-related reasons, and they also had a higher level of educational attainment than the Finnish general population. It is possible that the negative findings about the associations between affective symptoms and binge drinking or daily smoking among Russian migrants could indicate better health among these participants, which could be explained in part by the “healthy migrant effect” and voluntary nature of migration (Kennedy et al., 2014; Salas-Wright et al., 2014). However, the possibility of underreporting substance use among Russian participants could, to some extent, also explain these results.

In contrast to Russian migrants, Somali and Kurdish participants could be considered as forced migrants. Elevated odds ratios for co-occurring affective symptoms/suicidal ideation and substance use were found only among Kurdish migrants. This is in concordance with previous literature reporting that, e.g., impaired mental health may predispose forced migrants to substance use (Horyniak et al., 2016). Forced migrants have often experienced severe adversities and stress in the country of origin, during the journey, and in the post-migration phase (Patel et al., 2017). This situation of involuntary migration could expose forced migrants to more intense stressors compared with voluntary migrants. According to neurobiological stress-vulnerability theories, exposure to severe stressors causes neurobiological and epigenetic modifications (McEwen, 2017; Zannas & West, 2014), and this could in part explain differences in risk factors for impaired mental health and substance use among forced migrants vs. voluntary migrants. According to our results, the current affective symptoms seem to contribute to the susceptibility for substance use, especially among Kurdish migrants. Alternatively, the differences in the associations between affective symptoms and substance use could be explained by differences in the social acceptability of substance use between the Kurds and Russians. Substance use is widely prohibited in the Middle East (AlMarri & Oei, 2009; Baasher, 1981; Hafeiz, 1995), and therefore it is possible that only the Kurds suffering from affective symptoms tended to drink alcohol, whereas drinking is socially acceptable among the Russians in general, contributing to higher prevalence of alcohol use regardless of affective symptoms.

The negative findings among Somali migrants could be explained by the low prevalence of alcohol, tobacco, and cannabis use among the participants, which is likely to result from the protective role of their culture and religion on lifestyle and habits of substance use (Al-Hamzawi et al., 2015; AlMarri & Oei, 2009; Baasher, 1981; Hafeiz, 1995). However, our results for Kurdish migrants, the majority of whom are also Muslim, highlight the versatility between migrant groups, and emphasize that the screening of substance use in healthcare and mental healthcare cannot be neglected due to presumptions of substance use habits that are based on culture (Haasen et al., 2008). Therefore, our results suggest the need for further qualitative research on substance use habits among migrant populations.

## Strengths and limitations

Our study provides population-based data, with a relatively high response rate, on three migrant groups in one European country (Finland). Population-based data and comparison with the general population reduce observation bias, as cultural and other variations between the population groups can be recognized in comparison with data where migrants are considered as one population or are grouped according to the continent of origin. Our study adds to the limited body of population-based research on substance use among migrants and its association to mental health. Previous literature on Kurdish migrants is scarce and this study decreases this information gap.

However, the results of the study need to be interpreted carefully, as comparative data is scarce and, despite the relatively good response rate, the number of observations is limited. Low attendance of Somali migrants to the health examination, causing low response rate to the HSCL-25, is likely to make the results concerning Somali migrants less reliable compared to those about population groups with higher response rates. The effects of underreporting and attrition among all the population groups are difficult to estimate, despite the use of sample weights. The possibility of underreporting of substance use needs to be considered, especially concerning the low prevalence of binge drinking among Russian origin participants and the low rates of substance use among Somali migrants (Bhopal et al., 2004).

In this study, we have refrained from using HSCL-25 subscales (depression and anxiety) and have used only the global score to describe both depressive and anxiety symptoms, as recommended (Kuittinen et al., 2016). We have applied the generally used cut-off point of 1.75 (Hollifield et al., 2002), which has also been used in previous studies that include refugees, asylum seekers, and migrants from Afghanistan, Iran, Iraq, and Somalia in the Netherlands, in Sweden, and in Finland (Gerritsen et al., 2006; Rask et al., 2018; Rask, Castaneda et al., 2015; Rask, Suvisaari et al., 2015; Tinghög & Carstensen, 2009; Tinghög et al., 2010). However, the cut-off score has not been validated for these populations, which can be considered a limitation. To address this limitation, we performed univariate analyses using the HSCL-25 score as a continuous variable, and no major differences in findings were detected.

A limitation concerning suicidal ideation is that it was measured only with a single item from the HSCL-25, which does not measure other levels of suicide risk. A more extensive measure of suicide risk, with a scale specific to suicidal tendencies, could have made the results even more significant. Other limitations of our study include the selected dichotomization of binge drinking and daily smoking, as the reference groups for these variables included alcohol users without binge drinking and occasional smokers as well. In addition, some measurements of socioeconomic background, such as economic situation, were measured by self-reported evaluation in the lack of more objective measures. Different methods of data collection could have caused method-specific variance. Exploring the use of other substances, such as khat, could have provided more detailed information on substance use among the Somali participants. Causality relations cannot be deduced from our cross-sectional survey, and our results apply to the migrant groups studied.

## Conclusion

Our results draw attention to the comorbidity of affective symptoms, suicidal ideation, and substance use, especially among Kurdish migrants, and highlight the need for further qualitative research. Compared with the other groups studied, the Kurdish migrants seem to be in an especially vulnerable situation. From a public health perspective, our results imply the need to raise awareness among migrants of the harms of substance use and of treatment possibilities. The findings of our study also emphasize that substance use needs to be screened in all healthcare and mental healthcare patients regardless of the country of origin, and this cannot be neglected due to presumptions of substance use habits that are based on culture.
